# Dr. Haighton

**Published:** 1823-04

**Authors:** 


OBITUARY.—Died, on the 22d instant, Dr. HAIGHTON, Lecturer on Midwifery in the Borough.

